# The impact of leachate from waste rubber particles on duckweed: growth, oxidative stress, and cell structure

**DOI:** 10.3389/fpls.2026.1923439

**Published:** 2026-10-05

**Authors:** Shulan Zhao, Yingyue Gao, Ziqi Wang, Yi Zhang, Li’an Duo

**Affiliations:** Tianjin Key Laboratory of Animal and Plant Resistance, College of Life Sciences, Tianjin Normal University, Tianjin, China

**Keywords:** cellular structure, duckweed, membrane integrity, oxidative stress, tire wear particles

## Abstract

Waste rubber particles, especially tire wear particles (TWPs), have become major contributors to microplastic pollution. Through rainwater and road runoff, TWPs can enter the water environment, posing potential threats to aquatic organisms. However, systematic research on the effects of waste rubber particles on duckweed remains limited. Thus, *Spirodela polyrhiza* was exposed to rubber leachate at 10%, 15%, 20%, and 25% levels, aiming to evaluate the effects of rubber leachates on duckweed at the individual, physiological, and cellular levels. The results showed that rubber leachates significantly inhibited duckweed growth and reproduction. Exposure to the 25% leachate significantly reduced root length, root volume, and root activity of duckweed plants by 74.6%, 63.9%, and 29.7% (*p* < 0.05), respectively. Leachate exposure also induced the accumulation of osmoprotectants, with significant increases of soluble sugars (170.7%, *p* < 0.05) and proline content (73.6%, *p* < 0.05) under the 25% treatment. This was accompanied by reactive oxygen species (ROS) overproduction, reflected in significantly elevated superoxide anion and hydrogen peroxide contents in root tips. Furthermore, the leachates induced oxidative stress in duckweed, as evidenced by increased antioxidant enzyme activities and antioxidant contents. In addition, the leachates compromised the integrity of both cell and mitochondrial membrane, reduced cell wall component contents, and induced apoptosis. Collectively, these findings demonstrate that the toxicity of rubber leachates is mediated through oxidative stress and cellular structural damage.

## Introduction

With the mass production and wide application of rubber products, the amount of waste rubber generated worldwide is increasing annually, with waste tire rubber accounting for approximately 80% of the total waste rubber (Lu et al., 2025). Annually, more than 1.6 billion tires are produced worldwide, with approximately 1 billion waste tires generated each year (Saputra et al., 2021; Mayer et al., 2024). The three-dimensional crosslinked structure of rubber and the presence of additives result in the high resistance of scrap tires to natural degradation, resulting in their presence as long-term waste. Scrap tires are piled in open spaces, not only occupying landfill space but also providing breeding grounds for mosquitoes, facilitating disease spread and fire accidents (Zhao et al., 2024a).

Tire wear particles (TWPs) are generated by the friction between the tire tread and the road surface during vehicle driving. On average, each passenger vehicle releases approximately 1.4 kg of TWPs into the environment each year (Boisseaux et al., 2024). Tiny TWPs float in the air (Rausch et al., 2022), whereas larger particles are deposited in the soil on both sides of the road and enter the water ecosystem with road runoff (Zhao et al., 2024b). TWPs are considered the main source of microplastics. Recent reviews have highlighted that microplastics are not inert pollutants but act as dynamic environmental stressors (Ansari et al., 2025). In addition to their basic components (rubber, carbon black, and silica), tires contain 5–10% additives (such as plasticizers, softeners, antioxidants, ozone inhibitors, vulcanization activators, and accelerators) to ensure their stability and durability (Ni et al., 2023). These additives are inevitably released into the water environment and are detrimental to aquatic organisms. Numerous studies have shown that rubber particles and their leachates release large amounts of heavy metals (Liu et al., 2022), including zinc (Zn), lead (Pb), chromium (Cr), cadmium (Cd), and copper (Cu), as well as organic substances such as polycyclic aromatic hydrocarbons (PAHs), benzothiazole, and aniline (Yang et al., 2022; Gao et al., 2026), into aquatic ecosystems. The toxicity of rubber leachates is related to the presence of these toxic metals and organic compounds (Wagner et al., 2018). Once rubber particles are ingested by aquatic animals, they can stimulate the defense system, block the gastrointestinal tract, interfere with food intake, affect the activities of these animals, and increase their mortality rates (LaPlaca and van den Hurk, 2020).

TWPs are a major source of environmental contamination, releasing complex mixtures of chemicals into water bodies (Vogel et al., 2024; Wang et al., 2025). Previous studies have established that TWPs induce toxicity in a range of aquatic organisms, manifesting as accumulation in tissues, growth inhibition, oxidative stress, membrane damage, and alterations in behaviors and gut microbial composition (Khan et al., 2019; Chang et al., 2023; Ni et al., 2023; Boisseaux et al., 2024; Le Du-Carrée et al., 2024; Liu et al., 2026). Despite this body of evidence, the vast majority of studies have focused on aquatic animals, leaving a critical gap in our understanding of the effects of TWPs on aquatic macrophytes. In addition, TWPs are ubiquitous in soil environments, and their influence on terrestrial plants is reported by some studies (Zhao et al., 2026). TWPs and their leachate inhibited plant growth and photosynthesis, induced oxidative damage, and altered soil microbial community (Liu et al., 2025; Wasnik et al., 2026). The smallest TWPs (100 μm) showed the strongest phytotoxicity and disrupted soil microbial function (Iqbal et al., 2026).

Aquatic macrophytes are key components of freshwater, playing essential roles in nutrient cycling, water purification, and habitat provision (Cui et al., 2023). Duckweed is a floating plant of the Araceae family with a life cycle mainly characterized by asexual reproduction. It rapidly proliferates in warm seasons and is widely present in water environments such as paddy fields, lakes, and ponds. Thus, it has been widely used as a model test species in aquatic toxicological research (Cui et al., 2023; Putar et al., 2025). Previous studies have shown that duckweed could be potentially used as a bioindicator of microplastic pollution in fresh waters (Kalčíková, 2020; Rozman and Kalčíková, 2022; Cui et al., 2023). While previous studies have documented the toxicity of TWPs and leachates in terrestrial plants (Liu et al., 2025; Wasnik et al., 2026; Zhao et al., 2026), their effects on aquatic macrophytes—particularly duckweed, a model organism for aquatic toxicity testing—remain largely unexplored. Moreover, most existing studies have focused on short-term exposures and bulk material effects, with limited mechanistic insight into the specific pathways of toxicity. To address these gaps, we conducted a comprehensive investigation of the phytotoxic effects of rubber leachates on duckweed, evaluating growth inhibition, physiological disruption, oxidative stress, cellular damage, and alterations in cell wall components. This integrated multi-biomarker approach provides a mechanistic framework for understanding the toxicity of rubber leachates in aquatic ecosystems, advancing beyond previous studies that have primarily focused on single endpoints or terrestrial species. We hypothesized that (1) rubber leachates would inhibit duckweed growth and photosynthetic performance in a dose-dependent manner; (2) oxidative stress, characterized by ROS accumulation and lipid peroxidation, would be a primary driver of toxicity; (3) cellular damage, including membrane disruption and electrolyte leakage, would accompany oxidative stress; and (4) alterations in cell wall composition would occur as a secondary effect of oxidative damage. By integrating these endpoints, this study provides the first mechanistic framework for understanding rubber leachate toxicity in aquatic plants.

## Materials and methods

### Experimental materials

The duckweed (*Spirodela polyrhiza* (L.) Schleid.) plants were purchased from a commercial source. Water medium was collected from a local pond in Tianjin Normal University, China and the duckweed plants were cultured in containers containing pond water. Crumb rubber with particle size of 1–2 mm was processed from waste tires by Henan Xinao Company, China. The surface of these particles was cleaned with deionized water and air-dried. The stock rubber leachate was obtained by extracting 500 g of rubber particles with 500 mL of pond water in the dark and shaking for 7 days at 25 °C. Different concentrations of working leachates were obtained by diluting the stock leachate with pond water.

### Experimental design

Four treatments were performed with different concentrations of crumb rubber leachates added to cylindrical glass containers. The leachate concentrations of 10%, 15%, 20%, and 25% were obtained by transferring 10 mL, 15 mL, 20 mL, and 25 mL of stock leachate and 90 mL, 85 mL, 80 mL, and 75 mL of pond water into culture containers, respectively. Based on our preliminary experiments, these concentrations were used to maximize sensitivity in detecting toxic responses. A control was set up with duckweed plants and 100 mL pond water, and each treatment was replicated three times (n=3). Fifty healthy duckweed plants with two fronds were placed in containers for cultivation. The average temperature of the environment was 24 °C, relative humidity was17%–50%, and the light: dark cycle was 14 h:10 h. Each parameter was measured after seven days of culture.

### Plant growth and morphological analysis

Five individuals of *S. polyrhiza* were taken from each replicate according to Ceschin et al. (2023). The length and width of the fronds and the area were photographed under a stereomicroscope (Nikon SMZ25, Japan). The duckweed fronds were cut, rinsed, and blotted dry to measure fresh weight (FW).

The longest root length of each individual plant was measured using a stereomicroscope (Nikon SMZ25, Japan). According to Archimede’s principle, root volume was measured using the water displacement method in a graduated glass cylinder (Pang et al., 2011). The roots were thoroughly cleaned, gently blotted, and immersed in the water of the measuring cylinder, and the increase in water volume was recorded. Subsequently, the cleaned and blotted roots were weighed to obtain FW. Root activity was determined by the 2,3,5-triphenyltetrazolium chloride (TTC) method (Sheng et al., 2009). In brief, 0.1 g of root samples was fully submerged in 5 mL of 0.4% TTC (Solarbio) and 5 mL of 66 nmol·L^-1^ (pH 7.0) phosphate-buffered solution (PBS) in the dark at 37 °C for 3 h. Subsequently, 2 mL of sulfuric acid (1 mol·L^-1^) was added immediately to terminate the reaction. The cut roots were placed in a test tube, and 10 mL of methanol was added to completely submerge the root tips. The tube was kept at room temperature for 24 h until the root turned white, and the absorbance was measured at 485 nm using a spectrophotometer (Shimadzu UV-2600i, Japan).

For determination of duckweed reproductivity, ten healthy duckweed plants with two fronds were placed in each container with above culture medium. Photographs were taken on days 1 and 7 and the newly grown fronds were recorded.

### Analysis of physiological parameters

For chlorophyll analysis, 0.2 g of fresh fronds were cut into pieces and soaked in 10 mL of acetone:anhydrous ethanol (V:V = 1:1) solution in the dark for 24 h until the fronds were completely decolorized. The absorbance of the supernatant was measured at 663 nm and 645 nm, and the chlorophyll a, chlorophyll b, and total chlorophyll contents (mg/g) were calculated according to Ceschin et al. (2023).

For assays of osmoprotectants, the contents of soluble sugar and soluble protein were assessed using anthrone colorimetric method (Cao et al., 2014) and coomassile brilliant blue method (Hrebid et al., 2025), respectively. Proline contents were determined using the ninhydrin test (Wang et al., 2022a). To detect reactive oxygen species (ROS), the fluorescent reagent 2’,7’-dichlorodihydrofluorescein diacetate (DCFH-DA, Sigma) was diluted with PBS at a ratio of 1:1000 to 10 μmol·L^-1^, and root tips 2–3 mm long were carefully cut off with a blade and stained with diluted fluorescent dye in the dark for 30 min. After they were washed with PBS 3 times, the fluorescence intensity of the root tips was observed under an upright fluorescence microscope (Leica DM5000B, Germany). To determine the superoxide anion (O_2_^−^) content, 0.2 g of the sample was homogenized in 0.8 mL of 50 mM phosphate buffer (pH 7.8) and centrifuged at 12,000 × g and 4 °C for 20 min. 0.5 mL of the supernatant was mixed with 1 mL of hydroxylamine hydrochloride (Adamas). After incubation at 25 °C for 1 h, 1.0 mL of 17 mM p-aminobenzenesulfonic acid (MREDA) and 1.0 mL of 7 mM α-naphthylamine (Leyan) were added. The samples were allowed to stand for 20 min, and the absorbance was measured at 530 nm. To determine the hydrogen peroxide (H_2_O_2_) content, 0.1 g of sample was homogenized in 1 mL of 0.1% trichloroacetic acid (TCA, Adamas) and centrifuged at 15,000 × g for 15 min at 4 °C. 1 mL of the supernatant was mixed with 1 m L PBS (pH=7.0) and 2 mL of potassium iodide (Leyan). After 60 min of incubation, the absorbance was measured at 390 nm with a spectrophotometer (Shimadzu UV-2600i, Japan). H_2_O_2_ content was calculated using a standard calibration curve (Hrebid et al., 2025).

Lipid peroxidation was estimated by measuring malondialdehyde (MDA) content using the thiobarbituric acid (TBA) colorimetric method (Hu et al., 2022). For oxidative stress assays, fresh plants (0.1 g) were ground in 1 mL 50 mM PBS (pH 7.8). The homogenate was centrifuged at 8,000 × g for 10 min at 4 °C. The supernatant was used for the determination of antioxidant enzymes. Superoxide dismutase (SOD) was determined according to the method of Rico et al. (2013), and one unit of SOD activity (U) was defined as the amount of enzyme that inhibited 50% of the photochemical reduction of nitro blue tetrazolium chloride (NBT, Solarbio). Peroxidase (POD) activity was determined using the guaiacol oxidation method (Zhang et al., 2022). The amount of enzyme that increased A_470_ by 0.01 per min was defined as one unit of POD activity (U). CAT activity was determined using a kit from Shanghai Yuanye Biotechnology Co., Ltd. Assay for CAT was carried out following the instructions of the manufacture, and the absorbance was measured at 240 nm. One unit of CAT activity (U) was defined as the amount of enzyme that reduced A_240_ by 0.1 per min. For determination of ascorbate peroxidase (APX), 0.1 g of fresh plants was put in an ice-cold mortar, and 0.8 mL of PBS was added. The mixture was ground in an ice bath, and the homogenate was centrifuged at 12,000 × g for 20 min. Then, 0.1 mL of enzyme extract was added 1.7 mL of 50 mM PBS (pH 7.0) containing 0.1 mM EDTA, 0.1 mL of 5mM ascorbate (AsA) and 0.1 mL of 20 mM H_2_O_2_. The absorbance at 290 nm was monitored, and one unit of APX activity (U) was defined as the amount of enzyme that oxidized 1 μmol of AsA per min. The content of reduced glutathione (GSH) was determined using an assay kit from Beijing Solarbio Technology Co., Ltd. Assay for GSH was carried out following the protocols in the assay kit. To determine the ascorbic acid (AsA) content, 0.1 g of fresh plants was homogenized in 5% TCA at a ratio of 1:5 (W:V) and centrifuged at 12000 × g and 4 °C for 10 min. One millimeter of supernatant was combined with 1 mL of 5% TCA and 1 mL of anhydrous ethanol and vigorously vortexed. Then, 0.5 mL of 0.4% H_3_PO_4_-ethanol, 1 mL of 0.5% BP-ethanol, and 0.5 mL of 0.03% FeCl_3_-ethanol were added to the mixture. After vortex, samples were incubated at 30 °C for 90 min, and the absorbance was recorded at 534 nm with a spectrophotometer (Shimadzu UV-2600i, Japan).

### Analysis of cell structure

The cell apoptosis of duckweed root tips was determined according to the methods of Fan et al. (2013). The root tips of duckweed were carefully cut with a blade, washed, placed on slides, and stained with a fluorescent dual-probe FDA-PI staining solution (Bestbio) for 5 min, after which the samples were rinsed with distilled water 3 times and covered by a cover slip. Apoptosis in the root tips was then observed under an upright fluorescence microscope (Leica DM5000B, Germany).

The integrity of the plasma membrane was detected using the Evans blue (EB) staining method. A 2 mm × 2 mm square was cut from a frond of duckweed, soaked in 2 mL of EB (Solarbio) staining solution in the dark for 15 min, and observed under a stereomicroscope (Nikon SMZ25, Japan). For determination of EB absorbance, fronds (0.01 g) were incubated in 0.25% EB solution at room temperature for 15 min, washed with distilled water 3 times, and soaked in distilled water overnight. The fronds were removed and extracted in 1 mL of 50% methanol or 1% sodium dodecyl sulfate solution in a water bath at 50 °C for 1 h. The absorbance was measured at 595 nm with a spectrophotometer (Shimadzu UV-2600i, Japan).

Membrane permeability was measured by assessing the electrolyte leakage. 0.1 g of the cut plant samples were incubated in 10 mL of deionized water for 24 h at room temperature. The initial electrical conductivity (R_1_) was recorded, followed by heating the samples in a boiling water bath for 30 min and determining final conductivity (R_2_). Electrolyte leakage was calculated as R_1_/R_2_ × 100% (Hrebid et al., 2025).

Mitochondria were extracted following the methods of Tonshin et al. (2003). The levels of mitochondrial membrane proteins were determined using the Coomassie brilliant blue method. The isolated mitochondria were suspended in 1 mL of buffer. The protein concentration of the suspension was adjusted to 0.3 mmol·L^-1^. The suspension was incubated at 20 °C for 2 min, after which the permeability of the mitochondrial membrane was measured at 540 nm. The isolated mitochondria were suspended in 0.2% bovine serum albumin (BSA, Bioss). The protein concentration of the suspension was adjusted to 0.5 mg·mL^-1^. The absorbances at 550 nm and 630 nm were measured, and the absorbance ratio was calculated as Cytc/a.

Cell wall components were extracted and fractionated according to Ren et al. (2020). The cell wall was sequentially extracted with boiling water, 4% KOH at 25 °C for 12 h, and 24% KOH at 25 °C for 12 h to measure the contents of pectin, hemicellulose I, hemicellulose II, and cellulose, respectively. To determine the pectin content, a galacturonic acid standard curve was prepared. 200 μL of pectin extract was added 1 mL of sulfuric acid containing 0.0125 mol borax and heated in a boiling water bath for 5 min. After cooling on ice, the mixture was added 20 μL 0.15% hydroxybiphenyl (Sigma-Aldrich), incubated for 20 min, and determined the absorbance at 520 nm. Similarly, a glucose standard curve was generated to measure the hemicellulose content. 200 μL of hemicellulose I or II extract was added 10 μL of 80% phenol (MREDA), 1 mL of sulfuric acid, and heated in a boiling water bath for 15 min. After cooling on ice, the absorbance was recorded at 490 nm. To assay the cellulose content, the precipitate (0.005 g) was mixed thoroughly in distilled water (0.5 mL) followed by adding sulfuric acid (750 µL) slowly and incubation in an ice bath for 30 min. After centrifugation at 8,000 r min^-1^ and 4 °C for 10 min, the supernatant was diluted 20 times with distilled water. The resulting solution (300 µL) was mixed with anthrone ethyl acetate (70 µL), sulfuric acid (630 µL). After incubation in a boiling water bath for 10 min, the absorbance was measured at 620 nm with a spectrophotometer (Shimadzu UV-2600i, Japan).

### Data analysis

All data are expressed as mean ± standard deviation (SD). Data conforming to normality assumptions were analyzed by one-way ANOVA using SPSS 27.0 software. The Duncan’s multiple range test was used to assess significant differences among treatments at the *p* = 0.05 level, and Origin 2021 was used for plotting.

## Results

### Effect of rubber leachates on duckweed growth

Rubber leachate exposure inhibited duckweed growth in a dose-dependent manner (**Table 1**). The most pronounced effect was a 74.6% and 63.9% (*p* < 0.05) reduction in root length and volume, respectively, at the highest concentration (25%). The leachate treatment significantly reduced biomass and root activity, with the most prominent effect at the highest leachate concentration.

**Table 1:** Effects of rubber leachates on the morphology and growth of *S. polyrhiza*.

Leachate concentration	Control	10%	15%	20%	25%
Frond length (mm)	4.19 ± 0.06a	4.14 ± 0.07a	3.87 ± 0.08b	3.79 ± 0.02b	3.51 ± 0.05c
Frond width (mm)	3.54 ± 0.15a	3.30 ± 0.09b	3.05 ± 0.11c	2.83 ± 0.06d	2.65 ± 0.12d
Frond area (mm^2^)	18.58 ± 1.31a	17.07 ± 1.15a	15.30 ± 0.35b	14.17 ± 1.10b	14.81 ± 0.05b
Root length (cm)	2.32 ± 0.09a	1.32 ± 0.05b	0.82 ± 0.11c	0.64 ± 0.05d	0.59 ± 0.02d
Root volume (cm^3^)	0.036 ± 0.001a	0.025 ± 0.002b	0.023 ± 0.001b	0.019 ± 0.002c	0.013 ± 0.001d
Frond FW (g)	0.276 ± 0.006a	0.262 ± 0.006b	0.253 ± 0.004b	0.241 ± 0.004c	0.217 ± 0.003d
Root FW (g)	0.048 ± 0.001a	0.044 ± 0.000b	0.043 ± 0.001b	0.041 ± 0.001c	0.039 ± 0.001d
Root activity (μg g^-1^ h^-1^)	113.8 ± 4.1a	95.8 ± 1.7b	89.2 ± 1.7c	84.7 ± 0.7d	80.0 ± 0.2e

Different lowercase letters indicate significant differences among treatments (*p* < 0.05), as determined by the Duncan’s test.

The morphology of the duckweed root system under light microscope is shown in **Figure 1**. In the control group, the roots were green with clearly visible conductive tissue. In contrast, roots treated with leachates appeared yellow, with the color deepening as leachate concentration increased. Furthermore, leachate-treated roots were more fragile than those of the control group.

**Figure 1:**
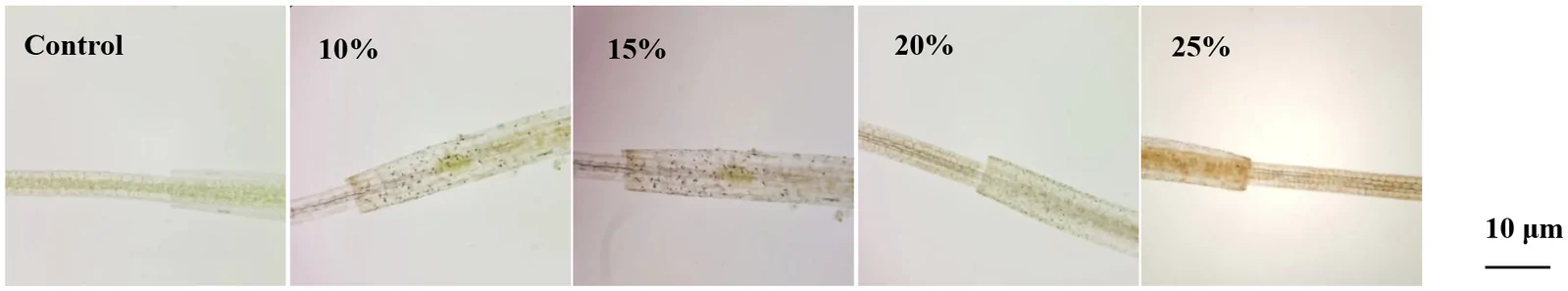
Effects of rubber leachate on root morphology of *S. polyrhiza*.

Rubber leachate exposure inhibited photosynthesis of duckweed by reducing chlorophyll content (**Figure 2**). Compared with the control, the maximum decrease in chlorophyll a and total chlorophyll content exceeded 30%. Additionally, the reproductivity of duckweed was significantly lower in leachate treatment groups than in the control (**Figure 2**). In the control group, the number of fronds increased by 50% from Day 1 to Day 7. In contrast, duckweed exposed to leachate produced only 1 to 3 additional fronds over the same period.

**Figure 2:**
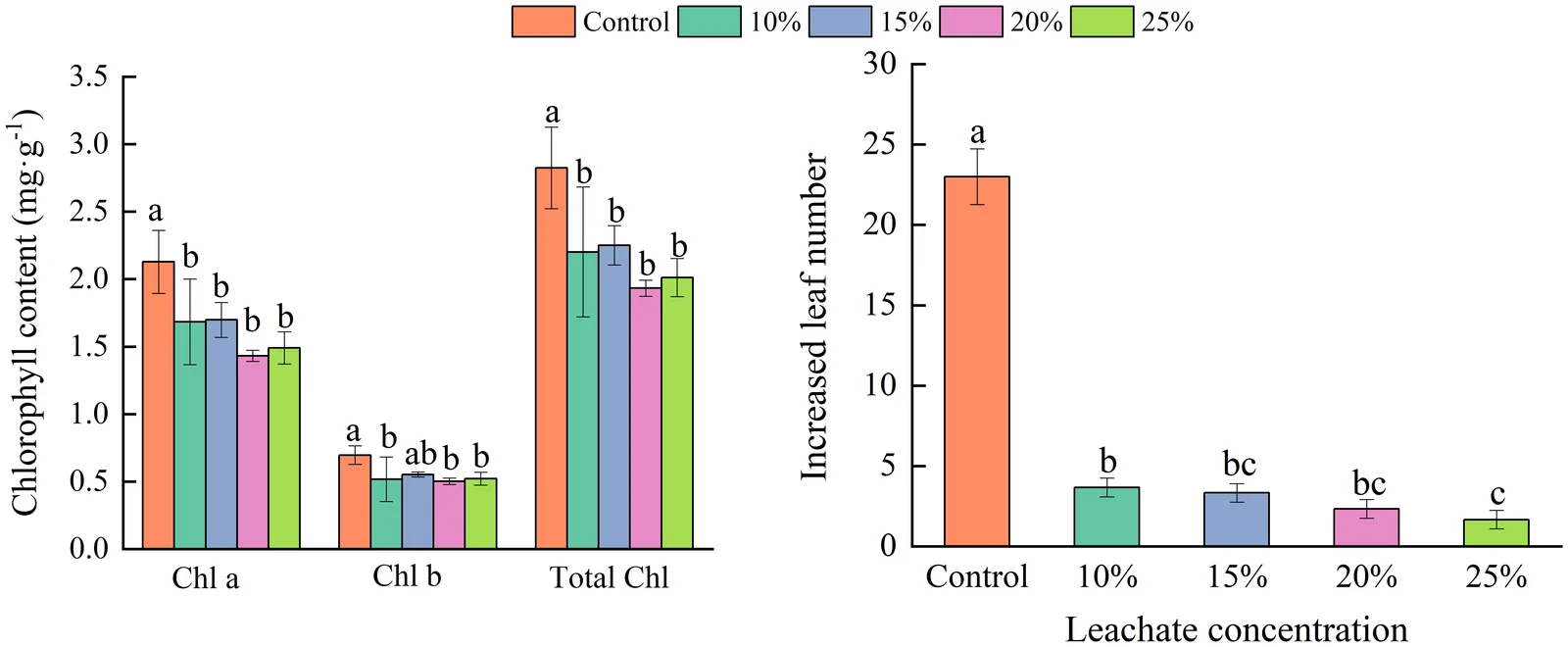
Effects of rubber leachate on chlorophyll contents and reproductivity of *S. polyrhiza*. Different lowercase letters above the error bars represent significant differences among treatments (*p* < 0.05), as determined by the Duncan’s test.

### Effects of rubber leachates on physiological characteristics of duckweed

The content of osmoprotectants soluble sugars, soluble proteins, and proline increased progressively with concentration (**Figure 3**). The most pronounced effect was a 170.7% and 73.6% (*p* < 0.05) increment in soluble sugars and proline content, respectively, at the highest concentration (25%).

**Figure 3:**
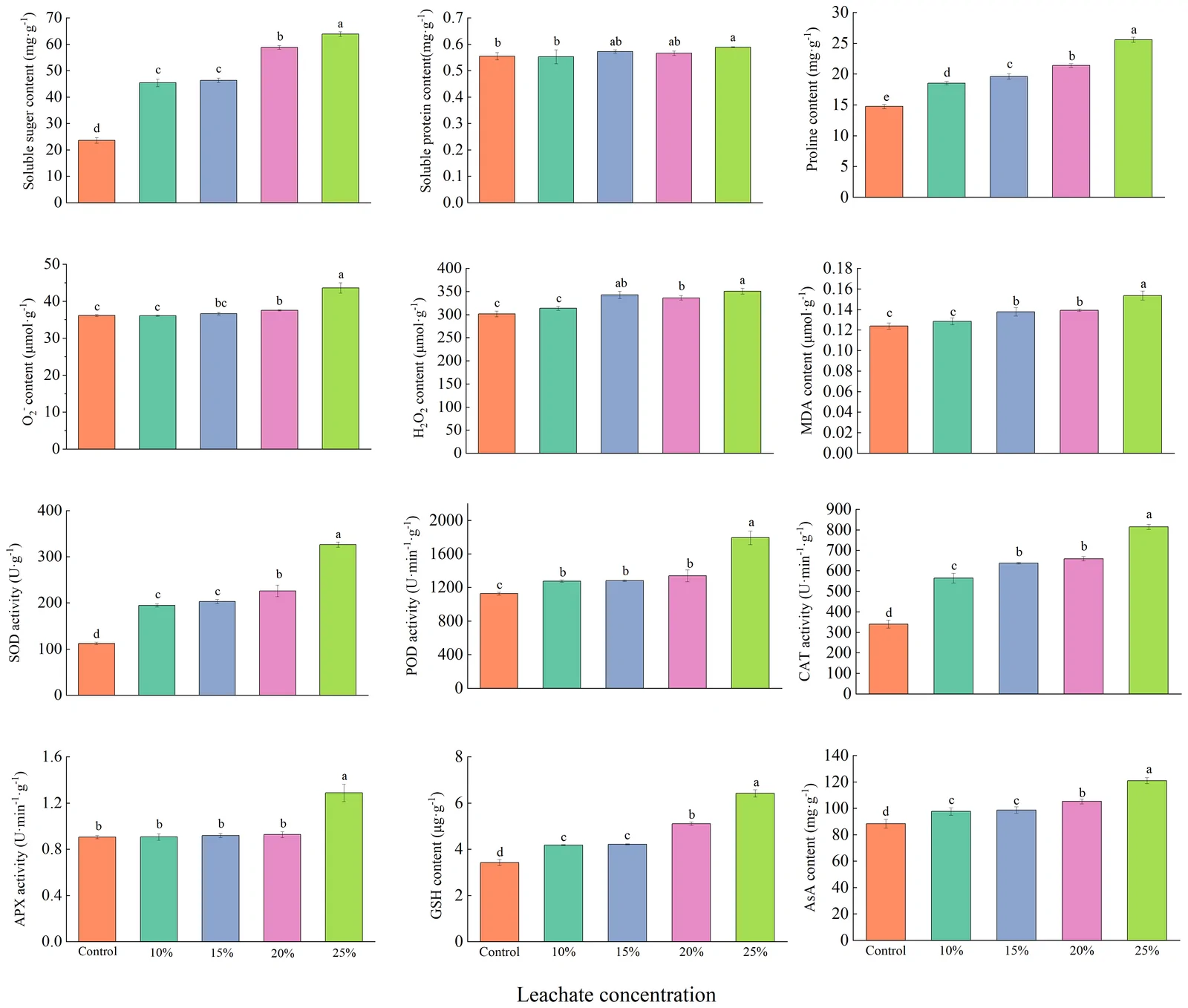
Effects of rubber leachate on physiological characteristics of *S. polyrhiza*. Different lowercase letters above the error bars represent significant differences among treatments (*p* < 0.05), as determined by the Duncan’s test.

Exposure to rubber leachates induced significant oxidative stress (**Figure 3**). O_2_^-^ and H_2_O_2_ content increased progressively with concentration. Lipid peroxidation, measured as MDA level, showed a similar dose-dependent increase, with the highest concentration showing a 24% increase compared to the control (*p* < 0.05). The activity of antioxidant enzymes (SOD, POD, CAT and APX) and the contents of antioxidants (GSH and AsA) showed the same response: increased progressively with concentration. Compared with the control, SOD and CAT activity increased by 190.8% and 139.4%, respectively, GSH content increased by 87.3%.

The fluorescence intensity of the ROS in the duckweed roots was detected using the fluorescent probe DCFH-DA (**Figure 4**). Fluorescence intensity was lowest in the control group. At leachate concentrations of 10% and 15%, ROS fluorescence intensity increased slightly. However, at 20% and 25% leachate concentrations, ROS fluorescence intensity rose significantly, indicating that ROS production and accumulation in duckweed increased in a concentration-dependent manner.

**Figure 4:**
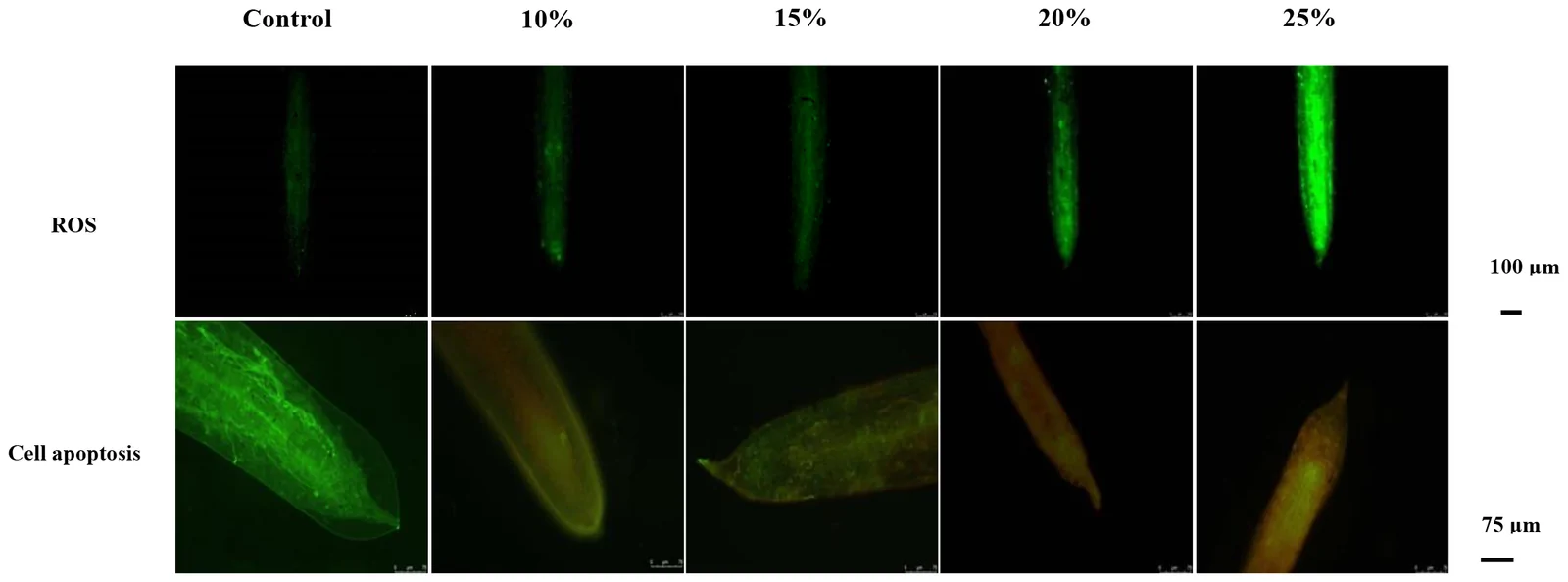
Effects of rubber leachate on fluorescence intensity of ROS and cell apoptosis in root tips of *S. polyrhiza*.

### Effect of the rubber leachates on duckweed cell structure

Cell apoptosis of the root tips of duckweed was observed by FDA-PI staining (**Figure 4**).

Root tip cells of duckweed in the control group exhibited bright green fluorescence, indicating that most cells were viable, with normal growth and high vitality. At 10% leachate concentration, a small proportion of root cells showed red staining, suggesting the onset of apoptosis. As leachate concentrations increased, the number of red-stained cells progressively rose. At the highest concentration (25%), the majority of root cells were stained red, indicating extensive apoptosis in root tips.

All the leachates significantly affected the integrity of the plasma membrane of the fronds of duckweed (**Figure 5**). In the control group, the plasma membranes of most duckweed leaf cells remained intact, as indicated by the absence of blue Evans blue (EB) staining. With increasing leachate concentration, the blue-stained area gradually expanded, reflecting a progressive rise in the number of cells with compromised membrane integrity. This damage was concentration-dependent, with higher leachate concentrations causing more extensive membrane disruption. Accordingly, EB content in the fronds increased in parallel with increasing leachate concentration.

**Figure 5:**
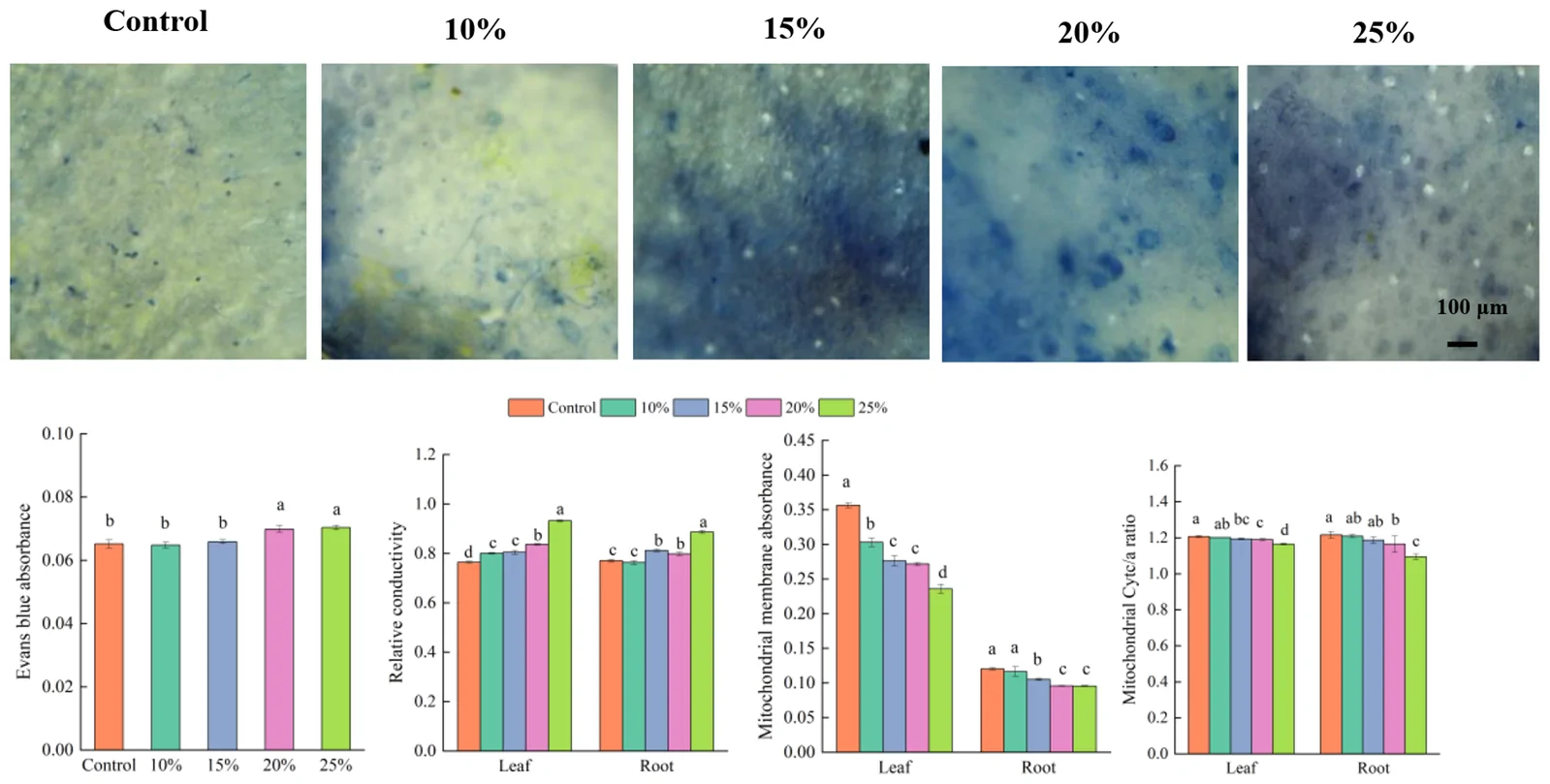
Effects of rubber leachate on cell membrane and mitochondrial membrane of *S. polyrhiza*. Different lowercase letters above the error bars represent significant differences among treatments (*p* < 0.05), as determined by the Duncan’s test.

An increase in plant cell electrical conductivity indicates damage or rupture of the cell membrane, which facilitates proton efflux and thereby elevates conductivity. As shown in **Figure 5**, the relative conductivity of both fronds and roots increased with higher leachate concentrations leading to progressively greater relative conductivity, indicating increasingly severe cell membrane damage.

Mitochondrial membrane protein absorbance in both roots and fronds was significantly lower in leachate-treated groups than in the control group (**Figure 5**). This reduction indicates that leachate exposure caused widening of mitochondrial membrane channels with absorbance of mitochondrial membranes decreasing. Additionally, the mitochondrial Cytc/a ratio in both fronds and roots declined significantly with increasing leachate concentration, reflecting a marked reduction in Cytc content.

The effects of leachate on duckweed cell wall components are presented in **Table 2**. Leachate exposure significantly reduced the contents of pectin, hemicellulose I, hemicellulose II, and cellulose in the cell walls of both fronds and roots in a concentration-dependent manner. At the highest concentration, the contents of pectin, hemicellulose I, and hemicellulose II in frond cell walls decreased by 58.5%, 72.7%, and 75.3% (*p* < 0.05), respectively, relative to the control. In root cell walls, the corresponding reductions were 52.9%, 45.8%, and 79.3%, respectively.

**Table 2:** Effects of rubber leachate on the components of the cell wall of duckweed (mg/g).

Treatments		Pectin content	Hemicellulose I content	Hemicellulose II content	Cellulose content
Leaf	Control	6.07±0.09a	13.02±0.29a	8.73±0.16a	59.46±0.81a
	10%	4.95±0.01b	8.22±0.12b	7.08±0.20b	60.83±1.46a
	15%	3.23±0.01c	4.85±0.25c	2.56±0.04c	55.85±1.39b
	20%	2.99±0.03d	3.88±0.10d	2.49±0.07cd	54.96±0.69b
	25%	2.52±0.001e	3.56±0.08d	2.16±0.15d	49.18±1.28c
Root	Control	4.86±0.02a	8.53±0.23a	7.24±0.06a	48.78±0.43a
	10%	4.26±0.03b	7.85±0.23b	6.91±0.07b	49.62±1.32a
	15%	3.25±0.05c	6.91±0.08c	2.08±0.14c	40.38±1.22b
	20%	2.81±0.10d	5.79±0.13d	1.80±0.07d	39.60±1.65b
	25%	2.29±0.06e	4.62±0.09e	1.50±0.07e	21.79±1.26c

Different lowercase letters indicate significant differences among treatments (*p*<0.05), as determined by Duncan’s multiple range test.

## Discussion

Root system condition is closely linked to overall plant growth and vigor (Gao et al., 2019). A well-developed root system enhances a plant’s capacity for nutrient absorption, transport, and accumulation, thereby promoting better growth. In duckweed, which lacks stems, the roots are particularly critical: they must not only absorb nutrients from the surrounding water but also efficiently translocate these nutrients to the fronds to support growth and development. In this study, leachate treatment significantly inhibited the growth of both roots and fronds. Specifically, root length, volume, and fresh weight were all markedly reduced, and the roots became fragile and prone to breakage, collectively impairing duckweed growth and development. Similarly, Le Du-Carrée et al. (2024) reported that leachate from TWPs inhibited algae growth and suggested that organic compounds in the leachate are largely responsible for the toxicity to the alga (Turner and Rice, 2010). Tire leachate has been shown to negatively affect *L. minor* growth, potentially due to nutrient deficiency arising from stunted root development (Pan et al., 2025). Shin et al. (2022) reported acute toxicity of TWP leachate to the rotifer *Brachionus plicatilis*, attributing the effect to a toxic cocktail of micro- and/or nano-sized TWPs and tire additives. Similarly, Dumont et al. (2023) characterized TWP leachate as a mixture of dissolved chemicals and nanoparticles, demonstrating additive toxicity. TWP leachates typically contain a complex mixture of metals—with Zn widely recognized as a primary driver of toxicity— organic compounds (e.g., PAHs, benzothiazoles), and transformation products (e.g., 6PPD-quinone) (Wik and Dave, 2009; Yang et al., 2022). Any or all of these constituents could contribute to the toxicity observed in the present study. However, without direct chemical analysis of our specific leachate, further speculation regarding the identity of the responsible toxicant(s) remains limited.

Photosynthetic pigments are key determinants of plant photosynthetic capacity and directly influence plant yield. In this study, leachate treatment significantly reduced chlorophyll content in duckweed, indicating that exposure to rubber leachate impaired photosynthetic capacity. Consistent with our results, Dumont et al. (2023) reported that dissolved chemicals in TWP leachate induced phytotoxicity in *L. minor*, with reductions of up to 50% in photosynthetic efficiency parameters. Similarly, Osman et al. (2025) observed that tire microplastics-derived leachates inhibited growth and photosynthetic pigment production in *S. polyrhiza*. These effects may be attributed to leachate-induced damage to root cell membranes and chloroplasts, or to impaired tetrapyrrole metabolism (Dalal and Tripathy, 2012).

Soluble sugars, soluble proteins, and proline are important osmoregulatory substances in plants, and their accumulation helps stabilize cell structure and function (Zhifang and Loescher, 2003; Ahanger et al., 2015). These osmolytes also contribute to stress resistance by enhancing cell water retention, maintaining membrane integrity, and preventing lipid membrane permeability changes, as well as enzyme denaturation and inactivation. In this study, leachate treatment significantly increased the contents of soluble sugars, soluble proteins, and proline in duckweed. Numerous studies have demonstrated that the accumulation of osmoregulatory substances is positively correlated with the degree of stress experienced by plant cells (Xin et al., 2020). Therefore, these results indicate that rubber leachate exerts stress on duckweed growth, with the stress intensity increasing with leachate concentration.

Under stress conditions, plants may generate excessive ROS, including primarily superoxide anions (O_2_•^-^), hydroxyl radicals (•OH), hydrogen peroxide (H_2_O_2_), and both radical and non-radical alkoxy species (Gill and Tuteja, 2010; Mandal et al., 2022). Excessive ROS accumulation can damage nucleic acids, proteins, and lipids by disrupting homeostasis (Potters et al., 2010). In this study, rubber leachates significantly increased ROS fluorescence intensity, O_2_•^-^ and H_2_O_2_ contents, and apoptosis in root tips. ROS-induced oxidative stress can exacerbate apoptosis in plants, thereby inhibiting growth (Zhang et al., 2015). To mitigate ROS-induced oxidative stress, plants have evolved complex antioxidant defense mechanisms that maintain ROS homeostasis (Sharma and Kaur, 2018). Antioxidant enzymes and nonenzymatic antioxidants work together to eliminate excess ROS and prevent oxidative stress-induced damage (Nieves-Cordones et al., 2019; Couee et al., 2006). In this study, the activities of SOD, CAT, POD, and APX, as well as GSH, ASA, and MDA contents all increased with leachate concentration. Consistent with these findings, Song et al. (2025) reported that TWP leachate disrupted antioxidant enzyme activity and elevated MDA levels in *Danio rerio*. Similarly, Osman et al. (2025) found that tire microplastics-derived leachates altered antioxidant enzyme activities and lipid peroxidation levels, triggering oxidative stress. Collectively, these results identify oxidative stress as a key mechanism of TWP leachate toxicity (Shin et al., 2022; Jiang et al., 2023). The observed oxidative stress response can be mechanistically attributed to the hazardous organic compounds and heavy metals present in the rubber leachate. Benzothiazoles and other aromatic compounds can undergo redox cycling, generating O_2_•^-^ and subsequently H_2_O_2_ and •OH. Zinc and organic additives can disrupt mitochondrial electron transport chain, leading to electron leakage and increased ROS production (Wasnik et al., 2026). The central role of oxidative stress as a mechanism of microplastic toxicity is well-established, with ROS generation leading to lipid peroxidation, membrane damage, and mitochondrial dysfunction across a range of organisms (Ansari et al., 2025).

Because Evans blue cannot penetrate intact cell membranes, it is widely used to assess cell viability. When cells are damaged, EB enters and binds to proteins, staining them blue (Feng et al., 2021; You et al., 2021). In this study, duckweed fronds exposed to rubber leachate were stained blue, with the color intensifying as leachate concentration increased, indicating that rubber leachates damaged frond cells. Quantification of EB content in fronds further corroborated these observations. Under stress, ruptured cell membranes release cytoplasmic electrolytes (Ran et al., 2020; Yin et al., 2021). Consistent with this, rubber leachates significantly increased electrolyte leakage of fronds and roots, indicating membrane damage—a finding that aligns with the elevated MDA levels observed.

Mitochondria are important organelles in eukaryotic cells, playing key regulatory roles in cellular energy metabolism, biosynthesis, and cell death, including apoptosis (Scheffler, 2001; Andersson and Kurland, 1999). ROS-induced oxidative stress can disrupt mitochondrial ultrastructure and inhibit energy synthesis, ultimately causing cell death in root tips (Xiao et al., 2019). When mitochondrial integrity is compromised, cytochrome c (Cytc) detaches from the inner membrane, leading to decreased Cytc content and Cytc/a ratio. In this study, leachate exposure damaged mitochondria in both fronds and roots, increased membrane permeability, and decreased the Cytc/a ratio. This aligns with our previous observation that rubber leachates exert toxicity on mitochondrial membrane and root cells in ryegrass (Duo et al., 2025). The cell wall is the first barrier encasing the plant cell, providing structural support and protection against biotic and abiotic stress (Bhandari et al., 2025). It comprises polysaccharides (pectin, cellulose and hemicellulose), lignin, and proteins, forming a multilayered adhesive structure that sustains cell growth, fixes cell shape and enhances mechanical strength (Delmer et al., 2024; Debnath et al., 2026). Studies have shown that abiotic stress can alter both the components and structure of plant cell walls; for example, lignin content often increases while pectin, hemicellulose, and cellulose contents decrease, leading to cell wall hardening as a stress resistance response (Wang et al., 2022b). In this study, leachate exposure significantly reduced the contents of pectin, hemicellulose, and cellulose in the cell wall, indicating that rubber leachates adversely affect cell wall composition and structure. The observed alterations in cell wall composition and membrane integrity can be mechanistically linked to oxidative stress and mitochondrial dysfunction. ROS attack polyunsaturated fatty acids in membrane lipids, initiating lipid peroxidation chain reactions (Duo et al., 2025). ROS can depolymerize cell wall polysaccharides, including cellulose, hemicellulose, and pectin, leading to altered cell wall composition and weakened structural integrity (Duo et al., 2025). In addition to chemical damage, rubber leachate constituents can directly interact with membrane lipids, altering membrane fluidity and causing depolarization of the plasma membrane potential (Michelangeli et al., 2025). Persistent membrane damage, coupled with mitochondrial dysfunction, can activate apoptotic pathways, leading to cell death.

Our findings have important implications for understanding the ecological risks of tire wear particles (TWPs) in aquatic ecosystems. While our study employed controlled laboratory exposures, the environmental reality is that TWPs are continuously released into waterways through road runoff, stormwater discharge, and atmospheric deposition, resulting in chronic, low-level exposure of aquatic organisms (citation). Unlike acute toxicity events, chronic exposure to sublethal concentrations can produce cumulative effects that may not be immediately apparent but can significantly impair organism fitness over time. Our study provides a mechanistic foundation for understanding the phytotoxicity of rubber leachates in duckweed. Future research should prioritize field validation, multi-generational and long-term exposure, chemical characterization and mixture effects, mitigation and remediation strategies.

## Conclusions

This study provides evidence that leachates from crumb rubber exhibit general toxicity to aquatic plants. The findings demonstrated that rubber leachates negatively affected plant growth and photosynthesis of duckweed. The effects increased with an increase in leachate concentration, which might be attributed to ROS generation. Furthermore, rubber leachates induced oxidative stress and damaged cell structure in duckweed. The negative effects of rubber leachates on *S. polyrhiza* might have been attributed to leachable chemicals from crumb rubber or oxidative stress. Thus, the limitations of this study and the necessity of future research were mainly related to the lack of quantitative analyses of leachable chemicals from crumb tire rubber.

## Data Availability

The original contributions presented in the study are included in the article/**Supplementary Material**. Further inquiries can be directed to the corresponding author.

## References

[B1] Ahanger M. A. Agarwal R. M. Tomar N. S. Shrivastava M. (2015). Potassium induces positive changes in nitrogen metabolism and antioxidant system of oat (*Avena sativa* L. cultivar Kent). J. Integr. Plant Interact. 10, 211–223. doi: 10.1080/17429145.2015.105626037339054

[B2] Andersson S. G. E. Kurland C. G. (1999). Origins of mitochondria and hydrogenosomes. Curr. Opin. Microbiol. 2, 535–541. doi: 10.1016/s1369-5274(99)00013-2 10508728

[B3] Ansari I. Arora C. Verma A. Mahmoud A. E. D. El-Kady M. M. Rajarathinam R. . (2025). A critical review on biological impacts, ecotoxicity, and health risks associated with microplastics. Water Air Soil Pollut. 236, 374. doi: 10.1007/s11270-025-07999-930311153

[B4] Bhandari D. D. Kim S. J. Brandizzi F. (2025). Fortifying the frontier: cell wall modifications during plant immunity. Curr. Opin. Plant Biol. 88, 102816. doi: 10.1016/j.pbi.2025.102816 41138697

[B5] Boisseaux P. Rauert C. Dewapriya P. Delignette-Muller M. L. Barrett R. Durndell L. . (2024). Deep dive into the chronic toxicity of tyre particle mixtures and their leachates. J. Hazard. Mater. 466, 133580. doi: 10.1016/j.jhazmat.2024.133580 38295724

[B6] Cao Y. Yang M. Li X. Zhou Z. Wang X. Bai J. (2014). Exogenous sucrose increases chilling tolerance in cucumber seedlings by modulating antioxidant enzyme activity and regulating proline and soluble sugar contents. Sci. Hortic. 179, 67–77. doi: 10.1016/j.scienta.2014.09.01642574925

[B7] Ceschin S. Mariani F. Lernia D. D. Venditti I. Pelella E. Iannelli M. A. (2023). Effects of microplastic contamination on the aquatic plant *Lemna minuta* (Least Duckweed). Plants 12, 207. doi: 10.3390/plants12010207 36616336 PMC9823687

[B8] Chang J. Jiao M. Zhang Z. Liu W. Li W. Xu P. . (2023). Mechanistic insight into the adverse outcome of tire wear and road particle leachate exposure in zebrafish (*Danio rerio*) larvae. Environ. Int. 178, 108053. doi: 10.1016/j.envint.2023.108053 37356306

[B9] Couee I. Sulmon C. Gouesbet G. (2006). Involvement of soluble sugars in reactive oxygen species balance and responses to oxidative stress in plants. J. Exp. Bot. 57, 449–459. doi: 10.1093/jxb/erj027 16397003

[B10] Cui R. Kwak J. An Y. J. (2023). Multigenerational effects of microplastic fragments derived from polyethylene terephthalate bottles on duckweed *Lemna minor*: Size-dependent effects of microplastics on photosynthesis. Sci. Total Environ. 872, 162159. doi: 10.1016/j.scitotenv.2023.162159 36775180 PMC9918310

[B11] Dalal V. K. Tripathy B. C. (2012). Modulation of chlorophyll biosynthesis by water stress in rice seedlings during chloroplast biogenesis. Plant Cell Environ. 35, 1685–1703. doi: 10.1111/j.1365-3040.2012.02520.x 22494411

[B12] Debnath J. Morton R. N. Engelsdorf T. Gigli-Bisceglia N. (2026). Plant cell wall remodeling and peptide signaling under abiotic and biotic stress. Plant Commun. 7, 101741. doi: 10.1016/j.xplc.2026.101741 41612691 PMC13084100

[B13] Delmer D. Dixon R. A. Keegstra K. Mohnen D. (2024). The *plant cell* wall-dynamic, strong, and adaptable-is a natural shapeshifter. Plant Cell 36, 1257–1311. doi: 10.1093/plcell/koad325 38301734 PMC11062476

[B14] Dumont E. R. Gao X. Zheng J. Macairan J. Hernandez L. M. Baesu A. . (2023). Unraveling the toxicity of tire wear contamination in three freshwater species: From chemical mixture to nanoparticles. J. Hazard. Mater. 453, 131402. doi: 10.1016/j.jhazmat.2023.131402 37062096

[B15] Duo L. Ding M. Li Y. Wang Q. Zhao S. (2025). Toxicity evaluation of long-term crumb rubber leachate in ryegrass roots: Morphology, stress physiology and cell structure. Ecol. Indic. 177, 113764. doi: 10.1016/j.ecolind.2025.11376442574925

[B16] Fan G. Liu D. Lin Q. (2013). Fluorescein diacetate and propidium iodide FDA-PI double staining detect the viability of *Microcystis* sp. after ultrasonic irradiation. J. Food Agric. Environ. 11, 2419–2421. doi: 10.1234/4.2013.5059

[B17] Feng Q. Ou Y. B. Han Y. de Dios V. R. Wang L. Zhang Q. . (2021). The brassinosteroid biosynthesis enzyme gene PeCPD improves plant growth and salt tolerance in *Populus tomentosa*. Ind. Crop Prod. 162, 113218. doi: 10.1016/j.indcrop.2020.11321842574925

[B18] Gao C. Mordehay V. Sherman A. Hämmerle L. E. Hofmann T. Polubesova T. . (2026). Tire wear particles as a source of organic contaminants in the agro-environment: Release of tire wear-derived compounds and their plant uptake. Environ. Res. 303, 124681. doi: 10.1016/j.envres.2026.124681 42103252

[B19] Gao M. Yang Y. Song Z. (2019). Toxicity of cadmium to wheat seedling roots in the presence of graphene oxide. Chemosphere 233, 9–16. doi: 10.1016/j.chemosphere.2019.05.127 31163310

[B20] Gill S. S. Tuteja N. (2010). Reactive oxygen species and antioxidant machinery in abiotic stress tolerance in crop plants. Plant Physiol. Biochem. 48, 909–930. doi: 10.1016/j.plaphy.2010.08.016 20870416

[B21] Hrebid A. K. Hatamnia A. A. Mohammadi M. Ranjbar M. E. (2025). Exogenous salicylic acid induces endogenous SA biosynthesis, enhances osmolyte accumulation, antioxidant defense, and free radical scavenging to alleviate chilling injury in red bell pepper fruit. Plant Physiol. Biochem. 229, 110524. doi: 10.1016/j.plaphy.2025.110524 40972329

[B22] Hu D. Li R. Dong S. Zhang J. Zhao B. Ren B. . (2022). Maize (*Zea mays* L.) responses to salt stress in terms of root anatomy, respiration and antioxidative enzyme activity. BMC Plant Biol. 22, 602. doi: 10.1186/s12870-022-03972-4 36539687 PMC9764725

[B23] Iqbal H. Baig A. M. Shi X. Shi R. Zeb A. Khan S. . (2026). Tire wear particles inhibit tomato growth and disrupt rhizosphere microbial function. J. Agric. Food. Chem. 74, 8795–8806. doi: 10.1021/acs.jafc.6c00598 41784106

[B24] Jiang J. Chen Z. Liao X. Liu Q. Zhou J. Ou S. . (2023). Identifying potential toxic organic substances in leachates from tire wear particles and their mechanisms of toxicity to *Scenedesmus obliquus*. J. Hazard. Mater. 458, 132022. doi: 10.1016/j.jhazmat.2023.132022 37453356

[B25] Kalčíková G. (2020). Aquatic vascular plants – A forgotten piece of nature in microplastic research. Environ. Pollut. 262, 114354. doi: 10.1016/j.envpol.2020.114354 32193083

[B26] Khan F. R. Halle L. L. Palmqvist A. (2019). Acute and long-term toxicity of micronized car tire wear particles to *Hyalella azteca*. Aquat. Toxicol. 213, 105216. doi: 10.1016/j.aquatox.2019.05.018 31185428

[B27] LaPlaca S. B. van den Hurk P. (2020). Toxicological effects of micronized tire crumb rubber on mummichog (*Fundulus heteroclitus*) and fathead minnow (*Pimephales promelas*). Ecotoxicology 29, 524–534. doi: 10.1007/s10646-020-02210-7 32342294

[B28] Le Du-Carrée J. Palacios C. K. Rotander A. Larsson M. Alijagic A. Kotlyar O. . (2024). Cocktail effects of tire wear particles leachates on diverse biological models: A multilevel analysis. J. Hazard. Mater. 471, 134401. doi: 10.1016/j.jhazmat.2024.134401 38678714

[B29] Liu H. Liang J. Du X. Lee Y. Abdullah A. L. B. Tay Y. J. . (2026). Toxic effects of tire wear particle leachate on benthic fish *Misgurnus anguillicaudatus*: An integrated assessment of immunosuppression, metabolic disruption, and microbiome dysbiosis across concentration gradients. Environ. Pollut. 397, 127958. doi: 10.1016/j.envpol.2026.127958 41839435

[B30] Liu Y. Sui Y. Haider F. U. Li S. Mu P. Zhang P. . (2025). Tire wear particles (TWPs) induced toxicity in wheat (*Triticum aestivum* L.) by affecting osmotic regulation, redox homeostasis, photosynthesis, and microbial density. Ecotoxicol. Environ. Saf. 303, 118950. doi: 10.1016/j.ecoenv.2025.118950 40886602

[B31] Liu Y. Zhou H. Yan M. Liu Y. Ni X. Song J. . (2022). Toxicity of tire wear particles and the leachates to microorganisms in marine sediments. Environ. Pollut. 309, 119744. doi: 10.1016/j.envpol.2022.119744 35843451

[B32] Lu Y. Gao F. Wang Y. Fu Y. Li Z. Xie J. . (2025). Exogenous DBT and overexpression of *deoR* improved the waste tire rubber desulfurization ability of a newly isolated *Escherichia* sp. strain. J. Hazard. Mater. 499, 140088. doi: 10.1016/j.jhazmat.2025.140088 41086491

[B33] Mandal M. Sarkar M. Khan A. Biswas M. Masi A. Rakwal R. . (2022). Reactive oxygen species (ROS) and reactive nitrogen species (RNS) in plants–maintenance of structural individuality and functional blend. Adv. Redox Res. 5, 100039. doi: 10.1016/j.arres.2022.10003942574925

[B34] Mayer P. M. Moran K. D. Miller E. L. Brander S. M. Harper S. Garcia-Jaramillo M. . (2024). Where the rubber meets the road: Emerging environmental impacts of tire wear particles and their chemical cocktails. Sci. Total Environ. 927, 171153. doi: 10.1016/j.scitotenv.2024.171153 38460683 PMC11214769

[B35] Michelangeli M. E. Brandsma S. H. Margalef M. Kuehr S. Spanu D. Gomes T. (2025). Elastomer leachates modulate haemocytes responses in *Mytilus edulis*. Mar. Pollut. Bull. 219, 118298. doi: 10.1016/j.marpolbul.2025.118298 40554080

[B36] Ni X. Zhou H. Liu Y. Zhan J. Meng Q. Song H. . (2023). Toxic effects of tire wear particles and the leachate on the Chinese mitten crab (*Eriocheir sinensis*). Environ. Pollut. 335, 122354. doi: 10.1016/j.envpol.2023.122354 37567401

[B37] Nieves-Cordones M. Lopez-Delacalle M. Rodenas R. Martínez V. Rubio F. Rivero R. M (2019). Critical responses to nutrient deprivation: a comprehensive review on the role of ROS and RNS. Environ. Exp. Bot. 161, 74–85. doi: 10.1016/j.envexpbot.2018.10.03942574925

[B38] Osman D. M. Yuan W. K. Nyaga M. P. Monikh F. A. Yang Y. Y. (2025). Dual acute effects of tire microplastics and their leachates on *Spirodela polyrhiza* and phyllosphere bacteria. Water Res. 287, 124362. doi: 10.1016/j.watres.2025.124362 40816017

[B39] Pan Y. F. Rauert C. Schmidt S. Thomas K. V. (2025). Impact of tyre wear particles and tyre leachate on the freshwater angiosperm *Lemna minor*. Environ. Chem. 22, EN25061. doi: 10.1071/en2506138477348

[B40] Pang W. Crow W. T. Luc J. E. McSorley R. Giblin-Davis R. M. Kenworthy K. E. . (2011). Comparison of water displacement and WinRHIZO software for plant root parameter assessment. Plant Dis. 95, 1308–1310. doi: 10.1094/pdis-01-11-0026 30731688

[B41] Potters G. Horemans N. Jansen M. A. K. (2010). The cellular state in plant stress biology - A charging concept. Plant Physiol. Biochem. 48, 292–300. doi: 10.1016/j.plaphy.2009.12.007 20137959

[B42] Putar U. Turk K. Jung J. Kim C. Kalčíková G. (2025). The dual impact of tire wear microplastics on the growth and ecological interactions of duckweed *Lemna minor*. Environ. Pollut. 368, 125681. doi: 10.1016/j.envpol.2025.125681 39805466

[B43] Ran C. Gulaqa A. Zhu J. Wang X. Zhang S. Geng Y. . (2020). Benefits of biochar for improving ion contents, cell membrane permeability, leaf water status and yield of rice under saline-sodic paddy field condition. J. Plant Growth Regul. 39, 370–377. doi: 10.1007/s00344-019-09988-9

[B44] Rausch J. Jaramillo-Vogel D. Perseguers S. Schnidrig N. Grobety B. Yajan P. (2022). Automated identification and quantification of tire wear particles (TWP) in airborne dust: SEM/EDX single particle analysis coupled to a machine learning classifier. Sci. Total Environ. 803, 149832. doi: 10.1016/j.scitotenv.2021.149832 34525712

[B45] Ren C. Qi Y. Huang G. Yao S. You J. Hu H. (2020). Contributions of root cell wall polysaccharides to Cu sequestration in castor (*Ricinus communis* L.) exposed to different Cu stresses. J. Environ. Sci. 88, 209–216. doi: 10.1016/j.jes.2019.08.012 31862062

[B46] Rico C. M. Morales M. I. McCreary R. Castillo-Michel H. Barrios A. C. Hong J. . (2013). Cerium oxide nanoparticles modify the antioxidative stress enzyme activities and macromolecule composition in rice seedlings. Environ. Sci. Technol. 47, 14110–14118. doi: 10.1021/es4033887 24266714

[B47] Rozman U. Kalčíková G. (2022). The response of duckweed *Lemna minor* to microplastics and its potential use as a bioindicator of microplastic pollution. Plants 11, 2953. doi: 10.3390/plants11212953 36365405 PMC9658923

[B48] Saputra R. Walvekar R. Khalid M. Mubarak N. M. Sillanpaa M. (2021). Current progress in waste tire rubber devulcanization. Chemosphere 265, 129033. doi: 10.1016/j.chemosphere.2020.129033 33250228

[B49] Scheffler I. E. (2001). A century of mitochondrial research: achievements and perspectives. Mitochondrion 1, 3–31. doi: 10.1016/s1567-7249(00)00002-7 16120266

[B50] Sharma R. Kaur R. (2018). Insights into fluoride-induced oxidative stress and antioxidant defences in plants. Acta Physiol. Plant 40, 181. doi: 10.1007/s11738-018-2754-030311153

[B51] Sheng M. Tang M. Chen H. Yang B. Zhang F. Huang Y. (2009). Influence of arbuscular mycorrhizae on the root system of maize plants under salt stress. Can. J. Microbiol. 55, 879–886. doi: 10.1139/w09-031 19767861

[B52] Shin H. Sukumaran V. Yeo I. C. Shim K. Y. Lee S. Choi H. K. . (2022). Phenotypic toxicity, oxidative response, and transcriptomic deregulation of the rotifer *Brachionus plicatilis* exposed to a toxic cocktail of tire-wear particle leachate. J. Hazard. Mater. 438, 129417. doi: 10.1016/j.jhazmat.2022.129417 35779397

[B53] Song Q. Meng Q. Meng X. Chen J. Cong J. (2025). Comparative toxicological impacts of tire wear and latex particle leachates on zebrafish embryos: Chemical characterization, oxidative stress, and transcriptomic disruption. Comp. Biochem. Phys. C. 298, 110323. doi: 10.1016/j.cbpc.2025.110323 40812569

[B54] Tonshin A. A. Saprunova V. B. Solodovnikova I. M. Bakeeva L. E. Yaguzhinsky L. S. (2003). Functional activity and ultrastructure of mitochondria isolated from myocardial apoptotic tissue. Biochemistry 68, 875–881. doi: 10.1023/a:1025798931614 12948388

[B55] Turner A. Rice L. (2010). Toxicity of tire wear particle leachate to the marine macroalga, *Ulva Lactuca*. Environ. Pollut. 158, 3650–3654. doi: 10.1016/j.envpol.2010.08.001 20828903

[B56] Vogel A. R. Kolberg Y. Schmidt M. Kahlert H. von Tümpling W. (2024). Potential deterioration of chemical water quality due to trace metal adsorption onto tire and road wear particles - Environmentally representative experiments. Environ. Pollut. 359, 124571. doi: 10.1016/j.envpol.2024.124571 39032551

[B57] Wagner S. Hüffer T. Klöckner P. Wehrhahn M. Hofmann T. Reemtsma T. (2018). Tire wear particles in the aquatic environment: A review on generation, analysis, occurrence, fate and effects. Water Res. 139, 83–100. doi: 10.1016/j.watres.2018.03.051 29631188

[B58] Wang Y. Gui C. Wu J. (2022b). Spatio-temporal modification of lignin biosynthesis in plants: A promising strategy for lignocellulose improvement and lignin valorization. Front. Bioeng. Biotech. 10, 917459. doi: 10.3389/fbioe.2022.917459 35845403 PMC9283729

[B59] Wang Y. Wang J. Cao X. Qi W. Peng J. Liu H. . (2025). Tire wear particles in aquatic ecosystems: Current knowledge and future perspectives. TRAC-Trend. Anal. Chem. 183, 118095. doi: 10.1016/j.trac.2024.11809542574925

[B60] Wang W. Zhang C. Zheng W. Lv H. Li J. Liang B. . (2022a). Seed priming with protein hydrolysate promotes seed germination via reserve mobilization, osmolyte accumulation and antioxidant systems under PEG-induced drought stress. Plant Cell Rep. 41, 2173–2186. doi: 10.1007/s00299-022-02914-6 35974188

[B61] Wasnik S. Cheng S. Zhao X. Keang K. Zhang H. Cross J. S. (2026). Tire wear particle and leachate induce oxidative stress and reshape the rice rhizosphere soil system: Multi-omics evidence of tissue-specific phytotoxicity. J. Hazard. Mater. 513, 142458. doi: 10.1016/j.jhazmat.2026.142458 42214864

[B62] Wik A. Dave G. (2009). Occurrence and effects of tire wear particles in the environment: A critical review and an initial risk assessment. Environ. Pollut. 157, 1–11. doi: 10.1016/j.envpol.2008.09.028 18990476

[B63] Xiao C. Wang L. Hu D. Zhou Q. Huang X. (2019). Effects of exogenous bisphenol A on the function of mitochondria in root cells of soybean (*Glycine max* L.) seedlings. Chemosphere 222, 619–627. doi: 10.1016/j.chemosphere.2019.01.195 30731382

[B64] Xin J. Ma S. Zhao C. Li Y. Tian R. (2020). Cadmium phytotoxicity, related physiological changes in *Pontederia cordata*: antioxidative, osmoregulatory substances, phytochelatins, photosynthesis, and chlorophyll fluorescence. Environ. Sci. Pollut. Res. 27, 41596–41608. doi: 10.1007/s11356-020-10002-z 32691317

[B65] Yang K. Jing S. Liu Y. Zhou H. Liu Y. Yan M. . (2022). Acute toxicity of tire wear particles, leachate and toxicity identification evaluation of leachates to the marine copepod, *Tigriopus japonicus*. Chemosphere 197, 134099. doi: 10.1016/j.chemosphere.2022.134099 35219709

[B66] Yin A. Huang B. Xie J. Huang Y. Shen C. Xin J. (2021). Boron decreases cadmium influx into root cells of *Capsicum annuum* by altering cell wall components and plasmalemma permeability. Environ. Sci. Pollut. Res. 28, 52587–52597. doi: 10.1007/s11356-021-14441-0 34014486

[B67] You Y. Liu L. Wang Y. (2021). Graphene oxide decreases Cd concentration in rice seedlings but intensifies growth restriction. J. Hazard. Mater. 417, 125958. doi: 10.1016/j.jhazmat.2021.125958 34020354

[B68] Zhang S. Gao W. Cai K. Liu T. Wang X. (2022). Effects of microplastics on growth and physiological characteristics of tobacco (*Nicotiana tabacum* L.). Agronomy 12, 2692. doi: 10.3390/agronomy1211269230654563

[B69] Zhang D. Ren L. Chen G. Zhang J. Reed B. M. Shen X. (2015). ROS-induced oxidative stress and apoptosis-like event directly affect the cell viability of cryopreserved embryogenic callus in *Agapanthus praecox*. Plant Cell Rep. 34, 1499–1513. doi: 10.1007/s00299-015-1802-0 26104871

[B70] Zhao T. Li G. Lozano Y. M. Wang Y. Lehmann A. Du D. . (2026). Beyond microplastics: How tire wear particles influence plant performance. Environ. Sci. Technol. 60, 8294–8302. doi: 10.1021/acs.est.5c16398 41810736

[B71] Zhao S. Liu M. Meng X. Liu A. Duo L. (2024a). Waste rubber - black pollution reframed as a global issue: Ecological challenges and sustainability initiatives. Environ. Pollut. 356, 124291. doi: 10.1016/j.envpol.2024.124291 38823550

[B72] Zhao T. Zhang Y. Song Q. Meng Q. Zhou S. Cong J. (2024b). Tire and road wear particles in the aquatic organisms – A review of source, properties, exposure routes, and biological effects. Aquat. Toxicol. 273, 107010. doi: 10.1016/j.aquatox.2024.107010 38917645

[B73] Zhifang G. Loescher W. H. (2003). Expression of a celery mannose 6-phosphate reductase in *Arabidopsis thaliana* enhances salt tolerance and induces biosynthesis of both mannitol and a glucosyl-mannitol dimer. Plant Cell Environ. 26, 275–283. doi: 10.1046/j.1365-3040.2003.00958.x

