# Supplementary material for: The impact of leachate from waste rubber particles on duckweed: growth, oxidative stress, and cell structure
Source: Front Plant Sci. 2026 Oct 5;17:1923439. doi: 10.3389/fpls.2026.1923439 (PMC13677379; doi:10.3389/fpls.2026.1923439)
Supplement: Supplementary file 1 [file Table1.docx]

Supplemental Methods

1. **Total soluble sugar (TSS)**

The TSS content was assessed using using anthrone colorimetric method (Cao et al., 2014). 0.05 g plant samples were soaked into 5 mL of distilled water, boiled for 30 min, and centrifuged at 5000 × g for 10 min. 0.5 mL of the supernatant was transferred to a new test tube, 1.5 mL of distilled water was added, followed by the addition of 2.5 mL of anthrone reagent. The reaction mixtures were vigorously shaken and then heated in a boiling water bath for 1 min. After cooling, the absorbance at 630 nm was measured with a spectrophotometer (Shimadzu UV-2600i, Japan) and the TSS content was calculated using a sucrose standard curve (Yemm and Willis, 1954).

2. **Soluble protein**

The soluble proteins were assessed using coomassile brilliant blue method (Hrebid et al., 2025). 0.1 g of plant samples were grounded in liquid-N_2_, homogenized in 10 mL of phosphate buffer (pH 7.0) containing polyvinylpyrrolidone. 2 mL of homogenate was centrifuged at 5000×g for 10 min. The supernatant was added Bradford solution and determined the absorbance at 595 nm with a spectrophotometer (Shimadzu UV-2600i, Japan) using a bovine serum albumin standard curve (Bradford, 1976).

3. **Proline**

Proline contents were determined using the ninhydrin test ((Wang et al., 2022; Bates et al., 1973). Briefly, 100 mg of plant samples was homogenized in 1 mL of 3 % sulfosalicylic acid and centrifuged at 5,000×g for 10 min. 0.4 mL of the supernatant was reacted with 0.4 mL of glacial acetic acid and 0.8 mL of acid ninhydrin, heated in a boiling water bath for 1 h, and then cooled in an ice bath. The mixture was vigorously mixed with 0.8 mL toluene and absorbance was recorded at 520 nm with a spectrophotometer (Shimadzu UV-2600i, Japan). Proline concentration was calculated using a standard curve and expressed as μg g^− 1^ FW.

4. **MDA**

Lipid peroxidation was estimated by quantifying malondialdehyde (MDA) using the thiobarbituric acid (TBA) reaction (Hu et al., 2022). Approximately 0.1g of plant tissue was homogenized in 1 mL of 10 % trichloroacetic acid (TCA). Samples were vigorously vortexed before centrifugation at 4000 r min^-1^ for 10 min at 4 °C. 2 mL of the supernatant were mixed with 2 mL of 0.5 % TBA, and incubated in a boiling water bath for 30 min, followed by cooling on an ice bath for 5 min and centrifugation at 4000 r min^-1^ for 10 min at room temperature. The absorbance of the supernatant was measured at 450, 532, and 600 nm with a spectrophotometer (Shimadzu UV-2600i, Japan). MDA concentration was calculated using 6.452×(A_532_-A_600_)-0.559×A_450_ and results were converted into μmol g^-1^ FW.

5. **SOD**

SOD activity was determined by quantifying the amount of enzyme required to cause a 50% inhibition of NBT. A 3.0 mL reaction mixture including 50 mM phosphate buffer, 0.1 mM EDTA, 2.0 µM riboflavin, 13 mM methionine, 75 µM NBT, and 100 µL of the enzyme extract was illuminated for 20 min and the absorbance was determined at 560 nm with a spectrophotometer (Shimadzu UV-2600i, Japan). One unit of SOD activity (U) was defined as the amount of enzyme that inhibited 50% of the photochemical reduction of NBT.

6. **POD**

For assay of POD activity, a 3 mL reaction mixture included 200 mM phosphate buffer (pH 6.0), 2.75 mM hydrogen peroxide, 8 mM guaiacol, and 100 µL of the enzyme extract. Absorbance was recorded at 470 nm with a spectrophotometer (Shimadzu UV-2600i, Japan) at least 2 min per 30 s. The amount of enzyme that increased A_470_ by 0.01 per min was defined as one unit of POD activity (U).

7. **Cell wall extraction**

The cell wall was extracted according to Ren et al. (2020). fresh roots and fronds (0.2 g) were ground in liquid nitrogen, homogenized with 75% (V/V) ethanol for 20 min in an ice bath, and centrifuged at 10000×g for 10 min at 4 °C. The supernatant was removed and the precipitate was then homogenized and with precooled acetone, methanol: chloroform (1:1, V/V), and methanol for 20 min each at 4 °C, and centrifuged at 10000×g for 10 min at 4 °C. All supernatants were discarded and the remaining precipitate was freeze-dried and designated as the cell wall.

8. **Cell wall fractionation**

The cell wall components were fractionated into pectin, hemicellulose I, hemicellulose II, and cellulose (Ren et al., 2020). The pectin fraction was extracted from the cell wall (2 mg) three times with ultrapure water in a boiling water bath for 1 h, and the supernatants were collected. The residue was further extracted twice with 4% KOH (W/W) at room temperature for 12 h, followed by extraction with 24% KOH. The supernatants from the 4% KOH and 24% KOH extractions were the hemicellulose I and hemicellulose II, respectively. The final precipitate was designated as cellulose.

**References**:

Cao, Y., Yang, M., Li, X., Zhou, Z., Wang, X., and Bai, J. (2014). Exogenous sucrose increases chilling tolerance in cucumber seedlings by modulating antioxidant enzyme activity and regulating proline and soluble sugar contents. *Sci. Hortic.* 179, 67–77.

Yemm, E.W., and Willis, A.J. (1954). The estimation of carbohydrates in plant extracts byanthrone. *Biochem. J.* 57, 508–514.

Hrebid, A.K., Hatamnia, A.A., Mohammadi, M., and Ranjbar, M.E. (2025). Exogenous salicylic acid induces endogenous SA biosynthesis, enhances osmolyte accumulation, antioxidant defense, and free radical scavenging to alleviate chilling injury in red bell pepper fruit. *Plant Physiol. Biochem.* 229, 110524.

Bradford, M.N. (1976). A rapid and sensitive method for the quantitation of microgram quantities of protein utilizing the principle of protein-dye binding. *Anal. Biochem.*72, 248–254.

Wang, W., Zhang, C., Zheng, W., Lv, H., Li, J., Liang, B., et al. (2022). Seed priming with protein hydrolysate promotes seed germination via reserve mobilization, osmolyte accumulation and antioxidant systems under PEG-induced drought stress. *Plant Cell Rep.* 41, 2173–2186.

Bates, L.S., Waldren, R.P., and Teare, I.D. (1973). Rapid determination of free proline for water stress studies. *Plant Soil* 39, 205–207.

Hu, D., Li, R., Dong, S., Wang, J., Zhao, B., Ren, B., et al. (2022). Maize (*Zea mays* L.) responses to salt stress in terms of root anatomy, respiration and antioxidative enzyme activity. *BMC Plant Biol.* 22, 602.

Ren, C., Qi, Y., Huang, G., Yao, S., You, J., and Hu, H. (2020). Contributions of root cell wall polysaccharides to Cu sequestration in castor (*Ricinus communis* L.) exposed to different Cu stresses. *J. Environ. Sci.* 88, 209–216.
